# A Sepsis-related Diagnosis Impacts Interventions and Predicts Outcomes for Emergency Patients with Severe Sepsis

**DOI:** 10.5811/westjem.2017.7.34770

**Published:** 2017-09-26

**Authors:** Mitchell Kim, Taketo Watase, Karl D. Jablonowski, Medley O. Gatewood, Daniel J. Henning

**Affiliations:** University of Washington, Division of Emergency Medicine, Seattle, Washington

## Abstract

**Introduction:**

Many patients meeting criteria for severe sepsis are not given a sepsis-related diagnosis by emergency physicians (EP). This study 1) compares emergency department (ED) interventions and in-hospital outcomes among patients with severe sepsis, based on the presence or absence of sepsis-related diagnosis, and 2) assesses how adverse outcomes relate to three-hour sepsis bundle completion among patients fulfilling severe sepsis criteria but not given a sepsis-related diagnosis.

**Methods:**

We performed a retrospective cohort study using patients meeting criteria for severe sepsis at two urban, academic tertiary care centers from March 2015 through May 2015. We included all ED patients with the following: 1) the 1992 Consensus definition of severe sepsis, including two or more systemic inflammatory response syndrome criteria and evidence of organ dysfunction; or 2) physician diagnosis of severe sepsis or septic shock. We excluded patients transferred to or from another hospital and those <18 years old. Patients with an EP-assigned sepsis diagnosis created the “Physician Diagnosis” group; the remaining patients composed the “Consensus Criteria” group. The primary outcome was in-hospital mortality. Secondary outcomes included completed elements of the current three-hour sepsis bundle; non-elective intubation; vasopressor administration; intensive care unit (ICU) admission from the ED; and transfer to the ICU in < 24 hours. We compared proportions of each outcome between groups using the chi-square test, and we also performed a stratified analysis using chi square to assess the association between failure to complete the three-hour bundle and adverse outcomes in each group.

**Results:**

Of 418 patients identified with severe sepsis we excluded 54, leaving 364 patients for analysis: 121 “Physician Diagnosis” and 243 “Consensus Criteria.” The “Physician Diagnosis” group had a higher in-hospital mortality (12.4% vs 3.3%, P < 0.01) and compliance with the three-hour sepsis bundle (52.1% vs 20.2%, P < 0.01) compared with the “Consensus Criteria” group. An incomplete three-hour sepsis bundle was not associated with a higher incidence of death, intubation, vasopressor use, ICU admission or transfer to the ICU in <24 hours in patients without a sepsis diagnosis.

**Conclusion:**

“Physician Diagnosis” patients more frequently received sepsis-specific interventions and had a higher incidence of mortality. “Consensus Criteria” patients had infrequent adverse outcomes regardless of three-hour bundle compliance. EPs’ sepsis diagnoses reflect risk-stratification beyond the severe sepsis criteria.

## INTRODUCTION

Sepsis is defined as life-threatening organ dysfunction caused by a dysregulated host response to infection.^1^ In response to the high morbidity, mortality,^2^,^3^ and cost^1^,^4^ associated with sepsis, clinical recommendations have been developed to promote the early recognition and aggressive treatment of sepsis.^5^ In 2015 these recommendations were integrated into the Center for Medicare and Medicaid Services (CMS) sepsis quality measure, NQF# 0500, which mandates three- and six-hour care bundles for patients with severe sepsis. This measure includes all patients with an *International Classification of Diseases, Tenth Revision, Clinical Manageme*nt (*ICD-10*) diagnosis of “severe sepsis” or “septic shock,” as well as “sepsis,” if patients demonstrate two or more systemic inflammatory response syndrome (SIRS) criteria, new sepsis-related organ dysfunction, and suspected infection—the definition of severe sepsis in the 1992 Consensus guidelines.^6^,^7^

More patients meet criteria for sepsis than those who are assigned a categorical sepsis diagnosis by emergency physicians (EP).^8^ This discrepancy raises the possibility that EPs under-identify patients who could benefit from early and aggressive treatment, delaying time-sensitive care for these critically ill patients, and negatively affect patient outcomes in sepsis.^8^ However, it is also possible that these under-identified patients compose a lower risk strata within the cohort of severe sepsis patients, mitigating the benefits of aggressive care.

This study of patients meeting criteria for severe sepsis compares differences in the primary outcome of mortality, and secondary outcomes of adverse events and sepsis-specific ED interventions, based on the presence or absence of an EP-assigned sepsis-related diagnosis. Furthermore, it evaluates the association between completing the CMS-prescribed three-hour sepsis bundle and adverse outcomes among patients who met severe sepsis criteria but were not given a sepsis-related diagnosis.

## METHODS

### Study Design

This was a retrospective, observational study of emergency department (ED) patients meeting criteria for severe sepsis by the 1992 Consensus guidelines. We conducted the study over three months at two urban, academic EDs with 90,000 combined annual visits. This study was approved by the Human Subjects Committee of our institutional review board with a waiver from informed consent, and conformed to previously established guidelines for retrospective chart reviews.^9^

### Study Subjects

This study included all patients during the study period who potentially could have met CMS NQF#0500 SEP-1 inclusion criteria for having severe sepsis in the ED. We included patients that met all of the 1992 Consensus criteria to define severe sepsis in the ED, which required having two or more SIRS criteria, new sepsis-related organ dysfunction, and suspected infection.^6^ Consistent with the 1992 Consensus criteria,^7^ new sepsis-related organ dysfunction was defined as creatinine > 2.0 mg/dL, bilirubin > 2.0 mg/dL, INR > 1.5, platelets <100,000 cells/mm^3^, lactate > 2.0 mmol/L, or systolic blood pressure (SBP) < 90mm Hg during the ED stay. Patients with an EP-diagnosis of “severe sepsis” or “septic shock” were likewise included, similar to the current CMS NQF#0500 SEP-1 identification process, even if patients did not strictly meet the 1992 Consensus criteria. We excluded patients who were < 18 years old, transferred from another facility, and those transferred from a study ED to another facility.

**Population Research Health Capsule**

What do we already know about this issue?

Patients who meet 1992 criteria for severe sepsis often do not receive an ED-documented sepsis diagnosis.

What was the research question?

Does this discrepancy represent under-recognition and an opportunity to expand sepsis-related care?

What was the major finding of the study?

Severe sepsis patients not given a sepsis-related diagnosis had low rates of adverse outcomes despite less aggressive care.

How does this improve population health?

Emergency physicians risk stratify sepsis patients beyond consensus criteria; broadly implementing aggressive care based on the presence of sepsis criteria alone may not improve outcomes.

We used our institutional electronic health record to screen all patients who were seen in the ED for the presence of two or more SIRS criteria, new sepsis-related organ dysfunction, and the presence of infection during the ED stay during the period from March 1, 2015, to May 31, 2015. All vital signs were documented electronically by the nurses. We used these vital signs and laboratory studies from the ED stay to identify SIRS criteria and new organ dysfunction that occurred at any point from triage to in-hospital transfer or initiation of boarding status. Subsequently, patients with two or more SIRS criteria and evidence of organ dysfunction were manually reviewed to determine whether organ dysfunction was new, and if an infection was the perceived cause of meeting SIRS criteria in the ED. This review used data obtained after admission to the hospital if these data were felt to be relevant to the ED presentation (i.e., blood culture results). Each subject had a diagnostic review performed by one of two board-certified, attending EPs, and we used a 10% overlapped sample to assess inter-rater reliability (kappa = 0.86, 95% CI: 0.77 – 0.94). This high kappa value justified using a single review for each subject, and for the few diagnostic disagreements in the 10% overlapping sample, the principal investigator’s (PI) adjudication was used. Next, we reviewed all patients who presented to the ED during the study period and had an *ICD-10* discharge diagnosis of “sepsis,” “severe sepsis,” or “septic shock,” “present on arrival”—indicating that the conditions treated during hospitalization were present when the patient arrived at the hospital. All of the patients with EP diagnosis of “severe sepsis” or “septic shock” were included, even if they did not strictly meet the 1992 Consensus definition of severe sepsis, similar to the CMS NQF#0500 SEP-1 guidelines for identifying patients with severe sepsis.

### Data Collection

In accordance with previously published guidelines for retrospective chart reviews,^10^ all data abstractors were trained and directly supervised by the study PI. Abstractors used data abstraction forms with clear definitions of the abstracted variables. Abstractors were blind to the study hypothesis. Interrater reliability for abstracted data was not performed, although in the spirit of direct supervision, abstractors were able and encouraged to seek clarification regarding the data they abstracted.

Elements of the past medical history and medications were manually abstracted by two research assistants who were trained and supervised by the PI. Patient outcomes, including the administration and timing of vasopressors, non-elective intubation, and death, were abstracted by a third-year emergency medicine resident physician. Likewise, elements of the three-hour sepsis bundle (CMS-approved antibiotics, blood cultures before antibiotics, measurement of serum lactate levels, and intravenous [IV] fluid bolus of 30 cc/kg within three hours of presentation for lactate ≥ 4.0 mmol/L or SBP < 90 mmHg),^7^ and the presence of shock in the ED were manually abstracted by the resident physician. Shock was defined as persistent hypotension (systolic blood pressure < 90 mmHg) despite the 30 cc/kg IV fluid bolus, elevated lactate (≥ 4.0 mmol/L), or vasopressor administration. ED vital signs, hospital disposition, hospital and intensive care unit (ICU) length of stay, and timing of transfers to the ICU were electronically abstracted for each visit from the electronic hospital database.

### Outcomes

The primary outcome was in-hospital mortality. Secondary outcomes were ICU admission, transfer to ICU in less than 24 hours, vasopressor administration, non-elective intubation, and completed elements of the NQF#0500 SEP-1 three-hour sepsis bundle.

### Data Analysis

We grouped patients based on the documented EP diagnosis: “Physician Diagnosis” or “Consensus Criteria.” All patients were manually screened to determine if an EP diagnosed the patient with sepsis, severe sepsis, or septic shock. The “Physician Diagnosis” group included all patients within this cohort given a sepsis-related diagnosis by the EP. The “Consensus Criteria” group met all criteria for severe sepsis^6^ without being given a sepsis-related diagnosis by the EP. Although, the physician diagnoses of infection (i.e., pneumonia) and organ dysfunction (i.e., hypotension) may indicate the presence of severe sepsis, we limited the “Physician Diagnosis” group to patients explicitly given a sepsis diagnosis to be consistent with the CMS guidelines and avoid confusion based on interpretation (i.e., acute renal failure if creatinine change did not meet CMS criteria for the diagnosis).

We used Student’s t-test or Wilcoxon rank-sum test as appropriate to compare continuous variables between groups. We compared binary covariates, including baseline characteristics of the two cohorts, between groups using the chi-square test. The rates of the primary and secondary outcomes were compared between groups using the chi-square test as well. We compared the mortality between patients who met all CMS three-hour bundle criteria, stratified by the diagnostic group (“Physician Diagnosis” or “Consensus Criteria”) to determine whether completing the NQF#0500 SEP-1 recommended three-hour bundle in either group was associated with adverse patient outcomes. We performed data analysis using SAS v9.3 statistical software (SAS Institute Inc., Cary, NC).

## RESULTS

During the three-month study period, 23,551 patients presented to the study EDs, of which 418 were identified as having severe sepsis either by physician diagnosis, or by the 1992 Consensus guidelines. We excluded 54 patients (50 transferred from another hospital, three transferred to another hospital, and one patient < 18 years old), leaving 364 patients for analysis (Figure). Of these patients, 121 (33.2%) were assigned a sepsis-related diagnosis by a treating EP (“Physician Diagnosis”) and 243 (66.8%) were identified by the 1992 Consensus guidelines without an EP diagnosis of sepsis, severe sepsis, or septic shock (“Consensus Criteria”).

Table 1 displays the baseline characteristics and source of infection for each group. The “Physician Diagnosis” group was more likely to have bacteremia, while the “Consensus Criteria” group was more likely to have an uncommon infection source. The “Physician Diagnosis” group was generally younger, yet more likely to have dementia or an indwelling urinary catheter; otherwise, the rates of comorbidities were similar between groups.

On average, patients identified as severe sepsis by physician diagnosis demonstrated higher presenting heart rate and temperature (Table 2). The mean minimum SBP in the “Physician Diagnosis” patients was lower than the “Consensus Criteria” patients (93.7 mm Hg vs 101.5 mm Hg, P < 0.01), and their average minimum respiratory rate was higher (18 per minute vs 16.4 per minute, P < 0.01) compared to the “Consensus Criteria” group (Table 2). Certain aspects of the clinical presentation were associated with the physician assigning a sepsis-related diagnosis among patients who met criteria for severe sepsis. Notably, the “Physician Diagnosis” group was more likely to have hypotension, elevated lactate or shock, established independent predictors of mortality in patients with presumed sepsis,^11^–^15^ and the “Consensus Criteria” patients were more likely to have thrombocytopenia or hyperbilirubinemia.

Table 3 shows that the patients in the “Physician Diagnosis” group had a higher rate of organ dysfunction (P < 0.01), including more frequent incidence of elevated lactate (P < 0.01), and hypotension (P < 0.01). They were also more likely to have do-not-resuscitate orders documented in the ED, which may reflect a higher rate of comorbidities in this group (P = 0.01) (Table 1). Furthermore, the patients in the “Physician Diagnosis” group were more likely to have shock in the ED (Table 4).

### Interventions

The “Physician Diagnosis” group more frequently received care that satisfied all elements of the three-hour bundle in the ED compared to the “Consensus Criteria” group (52.1% vs 20.2%, P < 0.01) (Table 5). Similarly, each individual component of the three-hour sepsis package was performed more frequently in the “Physician Diagnosis” group.

### Outcomes

“Physician Diagnosis” patients had significantly higher rates of the primary outcome of mortality compared to the “Consensus Criteria” group (12.4% vs 3.3%, P < 0.01) (Table 6). Non-elective intubation, vasopressor administration, and ICU admission likewise occurred more frequently in the “Physician Diagnosis” group compared to the “Consensus Criteria” patients. Lastly, “Physician Diagnosis” patients were more likely to be transferred from the ward to the ICU within 24 hours (6.6% vs 1.7%, P = 0.02).

To evaluate the association between the NQF#0500 SEP-1 three-hour bundle and adverse outcomes, we performed a stratified analysis by group to assess whether the mortality seen in either group was associated with failure to complete the three-hour sepsis bundle (Table 7). In the “Physician Diagnosis” group, those receiving the complete three-hour bundle had higher rates of non-elective intubation, vasopressor administration, ICU admission, and death. However, these differences were not statistically significant. For patients in the “Consensus Criteria” group, the rates of non-elective intubation and ICU admission were higher in those receiving the complete three-hour bundle. The rates of death were low overall in the “Consensus Criteria” group, and were not statistically different between those who received the complete three-hour bundle and those who did not.

## DISCUSSION

This study suggests that, among patients meeting severe sepsis criteria, EPs assign a sepsis-related diagnosis and provide more sepsis-related care to patients with a higher severity of illness. Despite a higher compliance rate with the three-hour bundle completion, patients within the “Physician Diagnosis” group had a higher rate of in-hospital mortality, vasopressor administration, ICU admission from the ED, and transfer to the ICU from the hospital ward. These results suggest that physicians recognize a higher risk population among those meeting criteria for severe sepsis, potentially based in part on the 1992 Consensus definitions, and this risk stratification leads to more aggressive interventions in the ED.

Within the “Consensus Criteria” cohort, mortality and all secondary outcomes occurred more frequently in patients who received a completed three-hour sepsis bundle. Again, the association between adverse outcomes and completion of the three-hour bundle in this group suggests that clinicians are identifying and aggressively treating those with more severe disease among patients meeting severe sepsis criteria. Patients in the “Consensus Criteria” group who received less aggressive care had lower rates of morbidity and mortality in comparison and, similarly, suffered lower mortality rates compared to prior study populations with severe sepsis.^16^ In light of the risks of antibiotic overuse,^17^ aggressive volume resuscitation^18^ and the need for resource stewardship,^19^ encouraging more nuanced sepsis care may be more appropriate, as opposed to broadly initiating standard bundles across this heterogeneous cohort of patients.

A previous study by Nguyen et al. noted a discrepancy between the frequency and severity of sepsis diagnoses when comparing EP diagnoses and the 1992 Consensus definitions.^8^ In this study, the authors were concerned that under-diagnosis represented under-recognition of high-risk patients, potentially leading to delays in early, critical treatments. In our study, the “Consensus Criteria” cohort did receive fewer individual three-hour bundle interventions and received the entire three-hour bundle significantly less frequently than those given a sepsis diagnosis. Yet, among these “under-identified” and “under-treated” patients, the infrequency of adverse outcomes calls into question the potential gains available if aggressive sepsis care were mandated for all patients meeting severe sepsis criteria. Future iterations of the CMS guidelines may improve resource utilization by integrating physician gestalt into the process of identifying patients, especially in the ED setting where risk-stratification is intrinsic to the EP’s role. Furthermore, emergency clinicians may temper the temptation to administer care according to the CMS guidelines across the spectrum of severe sepsis, when the care is driven solely to meet guidelines and not based on the sense of clinical utility.

## LIMITATIONS

There are several limitations inherent in our study. It was performed at two large, academic referral centers both located in the same urban setting, potentially limiting geographic generalizability. Similarly, the patient population studied may not adequately represent patients seen in rural or community settings. Our population was comprised of patients who presented during the spring months, and the study may not represent variations in the presentation of septic patients or potential efficacy of sepsis treatments for seasonal diseases. While the act of designating a sepsis-diagnosis and initiating sepsis treatment could vary seasonally at academic institutions based on resident training, all patient care and diagnoses were supervised by an attending physician, making temporal trends in diagnosis and treatment unlikely.

In addition, our study was observational, retrospective, and based on a chart review. These charts were written by a variety of medical providers (resident and attending physicians, physician assistants, and nurse practitioners) with a possibility of misclassification bias. Furthermore, a physician’s medical decision-making process may incorporate consensus guidelines, and different providers may have used these consensus guidelines to a variable degree based on their training. Lastly, there may be inaccuracies in obtaining information related to patients’ medical comorbidities, and timing of interventions (blood cultures before antibiotics, IV fluids within three hours, etc.) as this information was obtained from what was recorded in the chart. However, it is unlikely that these errors would be systematic in a way that would bias the overall results of the study.

## CONCLUSION

EP-assigned sepsis diagnoses reflect more severe illness, with increased in-hospital mortality and adverse outcomes, compared to ED patients meeting severe sepsis criteria but not specifically diagnosed as such. Patients of ED clinicians who are not specifically identified as septic by diagnosis in the ED chart, and who do not receive a completed three-hour bundle, nevertheless have lower rates of adverse outcomes, suggesting a less-ill cohort.

## Figures and Tables

**Figure:**
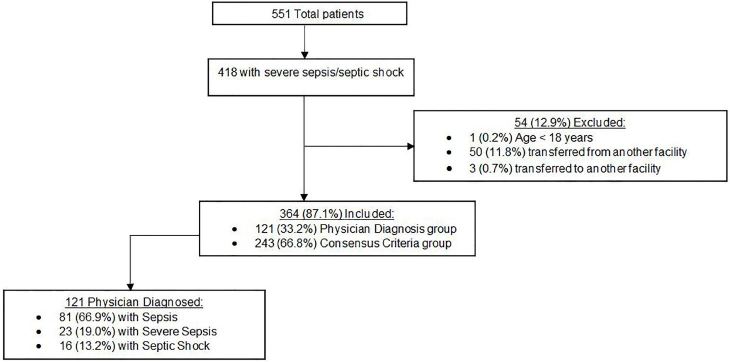
Study flow diagram of the total number of patients in this study of sepsis-related diagnosis, including the number of included and excluded patients.

**Table 1:** Demographics, comorbidities, and infectious source by group.

Characteristic	Physician diagnosis	Consensus criteria	p-value
N	121	243	
Age, mean in years (SD)	51.4 (17.4)	55.8 (17.4)	0.03*
Do not resuscitate # (%)	19 (16)	18 (7)	0.01*
Comorbidities # (%)			
None	8 (7)	21 (9)	0.5
Alcohol abuse	15 (12)	27 (11)	0.72
Urinary catheter	11 (9)	5 (2)	< 0.01*
Vascular catheter	5 (4)	7 (3)	0.53
Congestive heart failure	14 (12)	29 (12)	0.92
Coronary artery disease	15 (12)	22 (9)	0.32
Myocardial infarction	5 (4)	13 (5)	0.61
Chronic obstructive pulmonary disease	20 (17)	48 (20)	0.46
Other lung disease	13 (11)	20 (8)	0.43
Dementia	6 (5)	3 (1)	0.03*
Diabetes mellitus	36 (30)	58 (24)	0.23
Hypertension	55 (46)	85 (35)	0.05
Intravenous drug use	13 (11)	36 (15)	0.28
End stage liver disease	7 (6)	25 (10)	0.15
Chronic renal insufficiency	25 (21)	34 (14)	0.1
Hemodialysis	4 (3)	6 (3)	0.65
Stroke/transient ischemic attack	10 (8)	18 (7)	0.77
Solid malignancy	22 (18)	62 (26)	0.12
Hematologic malignancy	8 (7)	26 (11)	0.21
Human immunodeficiency virus	3 (3)	5 (2)	0.8
Metastatic cancer	7 (6)	18 (7)	0.56
Transplant	4 (3)	12 (5)	0.47
Infection source			
Urine	23 (19.0)	35 (14.4)	0.26
Pulmonary	46 (38.0)	77 (31.7)	0.23
Skin/soft tissue	26 (21.5)	48 (19.8)	0.7
Abdominal	11 (9.1)	39 (16.1)	0.07
Viral	5 (4.1)	18 (7.4)	0.23
Blood	21 (17.4)	17 (7.0)	< 0.01*
Other	15 (12.4)	56 (23.1)	0.02

*SD*, standard deviation.

Patients may have more than one infectious source identified during their stay.

* denotes statistical significance of p < 0.05.

**Table 2:** Vital signs of included patients.

Vital sign	Physician diagnosis	Consensus criteria	p-value
	
	Mean (SD)	Mean (SD)	
Initial SBP (mm Hg)	118.2 (28.2)	123.1 (26.2)	0.10
Initial DBP (mm Hg)	71.3 (20.2)	74.2 (18.2)	0.16
Initial temperature (°C)	37.4 (1.4)	37.1 (1.0)	0.01*
Initial HR (beats/minute)	110.7 (22.4)	105.8 (18.6)	0.03*
Initial RR (breaths/minute)	20.5 (5.6)	20.2 (7.2)	0.64
Initial oxygen saturation (%)	95.4 (6.3)	95.9 (4.8)	0.45
Maximum temperature (°C)	38.3 (1.5)	37.7 (1.2)	< 0.01*
Minimum temperature (°C)	36.6 (1.0)	36.5 (2.3)	0.53
Maximum HR (beats/minute)	112.8 (23.2)	114.9 (20.6)	0.38
Minimum HR (beats/minute)	93.8 (22.8)	86.9 (17.3)	< 0.01*
Maximum SBP (mm Hg)	135.4 (25.8)	136.9 (24.2)	0.59
Minimum SBP (mm Hg)	93.7 (20.2)	101.5 (21.1)	< 0.01*
Maximum RR (breaths/minute)	23.6 (7.1)	24.1 (9.3)	0.66
Minimum RR (breaths/minute)	18.0 (5.1)	16.4 (3.5)	< 0.01*

*HR*, heart rate; *DBP*, diastolic blood pressure; *RR*, respiratory rate; *SBP*, systolic blood pressure; *SD,* standard deviation.

* denotes statistical significance of p < 0.05.

**Table 3:** Frequency of organ dysfunction (OD) between the two groups of severe sepsis patients.

Organ dysfunction parameter	Physician diagnosis	Consensus criteria	p-value
Number of OD			< 0.01*
0	9 (7.4)	0 (0.0)	
1	54 (44.6)	158 (65.0)	
2	34 (28.1)	54 (22.2)	
3	12 (9.9)	22 (9.1)	
4	9 (7.4)	8 (3.3)	
5	2 (1.7)	0 (0.0)	
6	1 (0.8)	1 (0.4)	
Lactate > 2.0 mmol/L	82 (67.8)	125 (51.4)	< 0.01*
SBP < 90/MAP < 65 mmHg	59 (48.8)	67 (27.6)	< 0.01*
Creatinine > 2.0 mg/dL	28 (23.1)	42 (17.3)	0.18
Total bilirubin > 2.0 mg/dL	9 (7.4)	39 (16.1)	0.02*
Platelet < 100,000/uL	14 (11.6)	59 (24.3)	< 0.01*
INR > 1.5	18 (14.9)	38 (15.6)	0.85

*SBP,* systolic blood pressure; *MAP*, mean arterial pressure, *INR*, international normalized ratio.

* denotes statistical significance of p < 0.05.

**Table 4:** Determinants of shock in the emergency department.

Shock determinant	Physician diagnosis; number (%)	Consensus criteria; number (%)	p-value
Vasopressor administration	18 (14.9)	4 (1.7)	< 0.01*
Lactate > 4.0 mmol/L	11 (9.1)	11 (4.5)	0.09
SBP < 90 mmHg after 2L IVF	40 (33.1)	28 (11.5)	< 0.01*
Total with shock	52 (43.0)	38 (15.6)	< 0.01*

*IVF,* intravenous fluids; *SBP*, systolic blood pressure.

Note that patients may exhibit more than one criteria for shock.

* denotes statistical significance of p < 0.05.

**Table 5:** Three-hour bundle interventions received, by group.

Intervention	Physician diagnosis; number (%)	Consensus criteria; number (%)	p-value
Lactate checked	111 (91.7)	163 (67.1)	< 0.01*
Blood cultures before antibiotics	95 (78.5)	128 (52.7)	< 0.01*
Appropriate antibiotics given	72 (59.5)	74 (30.5)	< 0.01*
2L IVF given	72 (59.5)	73 (30.0)	< 0.01*
IVF not applicable	30 (24.8)	118 (48.6)	< 0.01*
Completed entire 3-hour bundle	63 (52.1)	49 (20.2)	< 0.01*

*IVF,* intravenous fluids.

* denotes statistical significance of p < 0.05.

** Includes patients where the three-hour bundle did not require IVF administration.

**Table 6:** The frequency and timing of adverse outcomes

Adverse event	Physician diagnosis; number (%)	Consensus criteria; number (%)	p-value
In-hospital mortality			
ED	0 (0.0)	2 (0.8)	0.32
Within 24 hrs	4 (3.3)	0 (0.0)	0.01*
24–72 hrs	4 (3.3)	3 (1.2)	0.18
>72 hrs	7 (5.8)	4 (1.7)	0.03*
Total	15 (12.4)	8 (3.3)	0.01*
Non-elective intubation			
ED	14 (11.6)	12 (4.9)	0.02*
Within 24 hrs	2 (1.7)	3 (1.2)	0.75
24–72 hrs	2 (1.7)	2 (0.8)	0.47
> 72 hrs	1 (0.8)	4 (1.7)	0.53
Total	19 (15.7)	21 (8.6)	0.04*
Vasopressor administration			
ED	19 (15.7)	4 (1.7)	< 0.01*
Within 24 hrs	4 (3.3)	10 (4.1)	0.71
24–72 hrs	3 (2.5)	1 (0.4)	0.07
>72 hrs	1 (0.8)	2 (0.8)	1.0
Total	27 (22.3)	17 (7.0)	< 0.01*
ICU admission			
From ED	53 (43.8)	43 (17.7)	< 0.01*
Within 24 hrs	8 (6.6)	4 (1.7)	0.02 *
24–72 hours	5 (4.1)	2 (0.8)	0.04 *
After 72 hours	1 (0.8)	6 (2.5)	0.43
Never	54 (44.6)	188 (77.4)	< 0.01 *

*ED,* emergency department; *ICU*, intensive care unit.

* denotes statistical significance of p < 0.05.

**Table 7:** Adverse outcomes, stratified by group, based on the completion of the entire 3-hour sepsis bundle.

Adverse oucome	Physician diagnosis; number (%)	p-value	Consensus criteria; number (%)	p-value
Death				
Completed 3hr bundle	10/63 (15.9)	0.23	3/49 (6.1)	0.21
Did not complete 3hr bundle	5/58 (8.6)		5/194 (2.6)	
Vasopressor administration				
Completed 3hr bundle	17/63 (27.0)	0.20	9/49 (18.4)	<0.01*
Did not complete 3hr bundle	10/58 (17.2)		8/194 (4.1)	
Non-elective intubation				
Completed 3hr bundle	13/63 (20.6)	0.12	8/49 (16.3)	0.03*
Did not complete 3hr bundle	6/58 (10.3)		13/194 (6.7)	
ICU admission				
Completed 3hr bundle	40/63 (63.5)	0.06	17/49 (34.7)	0.02*
Did not complete 3hr bundle	27/58 (46.6)		38/194 (19.6)	

*ICU*, intensive care unit.

* denotes statistical significance of p < 0.05.
